# Immunotherapy for Pediatric Leukemia

**DOI:** 10.3389/fonc.2013.00166

**Published:** 2013-07-01

**Authors:** Nirali N. Shah, Hema Dave, Alan S. Wayne

**Affiliations:** ^1^Pediatric Oncology Branch, National Cancer Institute, Center for Cancer Research, National Institutes of Health, Bethesda, MD, USA

**Keywords:** pediatric leukemia, immunotherapy, acute lymphoblastic leukemia, application of immunotherapy, adoptive cell therapy, monoclonal antibodies, hematologic malignancies

## Abstract

Substantial progress has been made in the treatment of leukemia in childhood. Despite this, leukemia remains a leading cause of pediatric cancer-related mortality and the prognosis is guarded for individuals with relapsed or refractory disease. Standard therapies are associated with a wide array of acute and long-term toxicities and further treatment intensification may not be tolerable or beneficial. The curative potential of allogeneic stem cell transplantation is due in part to the graft-versus-leukemia effect, which provides evidence for the therapeutic capacity of immune-based therapies. In recent years there have been significant advances in the development and application of immunotherapy in the treatment of leukemias, including the demonstration of activity in chemotherapy-resistant cases. This review summarizes immunotherapeutic approaches in the treatment of pediatric leukemia including current results and future directions.

## Introduction

Leukemia is the most common form of cancer in childhood. Acute lymphoblastic leukemia (ALL) accounts for approximately 75% and acute myelogenous leukemia (AML) about 20% of pediatric leukemia. Juvenile myelomonocytic leukemia (JMML) and Philadelphia chromosome positive chronic myelogenous leukemia (CML) are rare (Gloeckler et al., 1999). Despite great progress in the development of curative therapies, leukemia remains one of the most common causes of cancer-related mortality in pediatrics (Kaspers and Creutzig, 2005; Centers for Disease Control and Prevention, 2007; Lange et al., 2008; Hunger et al., 2012). Additionally, current therapies are associated with a high-risk of acute toxicities and long-term sequelae (Oeffinger et al., 2006; Armstrong et al., 2009; Diller et al., 2009). In recent years, there has been significant progress in the development and application of immunotherapy in the treatment of leukemias of childhood. Such immune-based therapies have the potential to overcome resistance to and decrease side effects of standard therapy. This review highlights immunotherapeutic approaches with applications in the treatment of pediatric leukemia.

## Allogeneic Stem Cell Transplantation and the Graft-*Versus*-Leukemia Effect: Potent Immunotherapy in the Treatment of Childhood Leukemia

Allogeneic hematopoietic stem cell transplantation (SCT) represents the most commonly employed and effective form of immunotherapy for childhood leukemia. SCT can be curative for all subtypes of pediatric leukemia and consensus guidelines for transplantation of acute leukemias have been published (Oliansky et al., 2007, 2012a,b). The *graft-versus-leukemia* (GVL) effect associated with allogeneic SCT is an immunologic reaction mediated by donor lymphocytes against foreign antigens expressed on leukemia cells. This GVL effect plays an important role in leukemia-free survival after transplantation (Marmont et al., 1991; Porter and Antin, 1999). Notably GVL may be linked with graft-versus-host disease (GVHD), a frequent complication of SCT caused by an immune reaction of donor cells against normal recipient tissues. Accordingly, relapse rates after matched sibling donor SCT are lower in individuals with AML and ALL who develop GVHD in comparison to those without GVHD (Sullivan et al., 1989; Horowitz et al., 1990). Furthermore, increasing the intensity of GVHD prophylaxis can reduce the GVL effect leading to a higher risk of relapse in children with acute leukemia. Consequently, the approach to SCT in children and adolescents with leukemia should attempt to balance the risks of GVHD with the potential benefits of GVL.

Despite the effectiveness of SCT and GVL in the treatment of leukemia, relapse remains the leading cause of treatment failure after SCT (Pavletic et al., 2010) and outcomes after post-transplant relapse are poor (Bajwa et al., 2012; Spyridonidis et al., 2012). Novel approaches are needed to reduce the risk of post-transplant relapse and to improve the outlook for individuals who relapse after SCT (Bishop et al., 2010, 2012).

### Donor lymphocyte infusions for post-transplant relapse

Donor lymphocytes infusions (DLI) can be curative for the treatment of post-transplant relapse, which provides further evidence of the potential of immunotherapy to cure leukemia. Complete remission (CR) rates of 70% or higher are observed in the setting of chronic phase CML, with lower rates (0–30%) in accelerated and blastic phase CML, ALL, AML, and JMML (Collins et al., 1997; Yoshimi et al., 2005; Tomblyn and Lazarus, 2008). Response rates are highest in the setting of molecular or cytogenetic relapse. The optimal use of DLI for pediatric patients remains incompletely defined. DLI are usually recommended for chronic phase CML, although *bcr/abl* kinase inhibitors are also effective (Wright et al., 2010). The results of DLI are poor for children with ALL and AML (Porter et al., 2010).

### Limitations of the GVL effect

Despite the clear evidence that an allogeneic effect contributes to the success of SCT for leukemia, as noted above, GVL is not uniformly effective. For example, in contrast to myeloid cells, B-lineage lymphoblasts often lack or have low-level expression of T cell co-stimulatory molecules such that ALL blasts present antigens poorly and may induce T cell anergy (Cardoso et al., 1996). Another potential obstacle is the rapid proliferative rate of most childhood leukemias, such that the tempo of progression may outpace the kinetics of DLI-induced GVL. Consequently, novel approaches to augment the GVL effect are needed. Additional general limitations of SCT include the need for a suitable stem cell donor, potential lack of donor availability for DLI (e.g., after umbilical cord blood transplants), the risks of GVHD and other causes of treatment-associated morbidity and mortality. For all of these reasons, extensive efforts are underway to develop alternative immunotherapeutic approaches.

## Adoptive Cell Therapies

The successes and the limitations of DLI in the management of post-transplant leukemic relapse have spurred investigations of other forms of adoptive cellular therapies, both in the allogeneic and autologous settings.

### Lymphocytes

Cytotoxic T-lymphocytes (CTLs) have the ability to recognize target antigens on malignant cells. Notably, healthy stem cell donors may have pre-existing leukemia-associated antigen-specific CTLs that can expand in transplant recipients, which may contribute to the eradication of leukemia after allogeneic transplantation (Rezvani et al., 2003). CTLs can be isolated from circulation, expanded *ex vivo* and infused to treat cancer. Several leukemia-associated antigen targets are being explored for CTL mediated therapy, for example the Wilms tumor-1 (WT1) gene product (Doubrovina et al., 2012a). WT1-specific T cells have been shown to correlate with relapse-free survival in patients with hematologic malignancies after allogeneic SCT (Rezvani et al., 2007). The use of CTLs is not limited to treatment of leukemia relapse. CTLs specific to Epstein–Barr virus (EBV), cytomegalovirus (CMV), and adenovirus are currently under investigation to treat or prevent viral reactivation (Leen et al., 2012) and EBV-lymphoproliferative disorders (Rooney et al., 1998; Bollard et al., 2008; Doubrovina et al., 2012b). CTLs directed against minor histocompatibility antigens have been successfully used to treat relapsed leukemia after SCT in adults (Falkenburg et al., 1999; Warren et al., 2010). There are also strategies that can be employed to enhance lymphocyte effector functions in general, for example, the *ex vivo* culture of lymphocyte subsets in specific conditions (Fowler and Gress, 2000; Porter et al., 2006). Limitations of adoptive T cell therapies include the need to identify target antigens with broad applicability, the potential requirement for human leukocyte antigen (HLA) matching, “off-target” toxicities such as GVHD and the possible need for long-term persistence of the transferred cells – all of which are being addressed in ongoing investigations.

### Natural killer cells

Natural killer (NK) cells are lymphocytes of the innate immune system that can mediate a potent GVL effect early after SCT (Ruggeri et al., 2008). Cytotoxicity is determined in part by mismatch in the NK cell killer immunoglobulin-like receptor (KIR) types between host and recipient, which are linked to the HLA system. Thus, allogeneic donors can be selected for NK cell KIR mismatch. A large number of clinical trials of allogeneic and autologous NK cells are being conducted for individuals with active leukemia or to augment GVL after SCT. NK cell cytotoxicity has been shown to be potent against AML cells (Murphy et al., 2012). Accordingly, the Children’s Oncology Group (COG) is conducting a trial designed to assess the impact of NK cell KIR mismatch in the setting of matched unrelated donor SCT for high-risk pediatric AML (NCT00553202). A St. Jude Children’s Research Hospital study of NK cells transduced with receptors to target CD19 is being conducted for children with ALL (NCT00995137). Results are pending. A clinical trial of donor-derived *ex vivo* activated NK cells administered following SCT for children, adolescents, and young adults with leukemias and solid tumors is in progress at the National Cancer Institute (NCI) (NCT01287104).

### Chimeric antigen receptors

T cells can be directed to target antigens on the surface of leukemia blasts by introducing chimeric antigen receptors (CARs) through gene therapy techniques. In general, the external domain of CAR constructs consists of a monoclonal antibody variable fragment (Fv), while the internal domains include components of the T cell receptor signaling and often a co-stimulatory signaling domain. CAR transduced cells are designed to enable immune effectors to bind to and induce cellular cytotoxicity against malignant cells that express target differentiation antigens (Cooper et al., 2003; Rossig et al., 2005; Lee et al., 2012a). CARs recognize native cell-surface antigens independently of antigen processing or HLA-restricted peptide fragment presentation, which differs from CTL based therapy. Most of the current CARs in testing utilize single chain variable domains (ScFv) derived from a mouse monoclonal antibody that binds to the target antigen, most commonly to date, CD19 for the treatment of B-lineage hematologic malignancies. The first generation CARs employed the CD3ζ cytoplasmic domain to elicit T cell responses, but subsequent constructs also include co-stimulatory molecules that serve to augment the T cell response. Trials of T cells and NK cells engineered with CARs directed to leukemia-targets are currently undergoing study in children and adults with ALL and non-Hodgkin lymphoma (NHL) at the Baylor College of Medicine (NCT00586391, NCT00608270, NCT00840853), NCI (NCT01593696, NCT01087294), Children’s Hospital of Philadelphia (CHOP) (NCT01029366), St. Jude Children’s Research Hospital (NCT00995137), and the University College, London (NCT01195480). Studies in adults demonstrate that CAR-based therapy can lead to sustainable remissions with CAR T cell expansion and persistence in the setting of chronic lymphocytic leukemia (CLL) and NHL (Kochenderfer et al., 2010, 2012; Porter et al., 2011). Early results in pediatric ALL are similarly encouraging, including the induction of minimal residual disease (MRD) negative CRs in cases of chemotherapy refractory disease (Curran et al., 2012; Lee et al., 2012b; Porter et al., 2012). CARs targeting other antigens such as CD22 and ROR1 in ALL (Baskar et al., 2012; Dave et al., 2012; Haso et al., 2012) and CD33 in AML (Dutour et al., 2012) are in development.

## Monoclonal Antibodies and Their Conjugates

Since monoclonal antibodies (MoAbs) were first generated against human differentiation antigens there has been the expectation that they would be used to treat hematologic malignancies (Kohler and Milstein, 1975). Myeloid and lymphoid leukemias are excellent candidates for MoAb-based therapies as they demonstrate surface expression of multiple human differentiation antigens that are rarely, if ever, found on other normal tissues and are generally accessible in the circulation. Importantly, due to the severe immunosuppression that is commonly associated with leukemia and its treatment, the incidence of anti-drug antibody formation (e.g., human anti-murine antibodies, anti-toxin antibodies) may be reduced. Advances in antibody engineering have led to the production of MoAbs and their fragments for clinical use and to generate humanized chimeric constructs to reduce their immunogenicity. Multiple reagents that target leukemia-associated surface antigens have been developed for clinical investigation (Table 1), some of which have applications in pediatric leukemia.

**Table 1:** **Monoclonal antibodies and conjugates**.

Antigen	Unconjugated monoclonal antibodies	Conjugates
CD2, CD3	Antithymocytes globulin, OKT3, siplizumab, UCHT1	Pokeweed antiviral protein, saporin, ricin-dgA, diphtheria toxin A, TRAIL
CD19	MDX-1342, BU-12	Maytansinoid DM4, liposomal doxorubicin, pokeweed antiviral protein, ricin-dgA, ricin-blocked saporin, ^131^I, ^90^Y
CD19 and CD3 (bi-specific)	Blinatumomab	–
CD20	Rituximab, ofatumumab	Calicheamicin, ^131^I, ^111^In, ^90^Y
CD19 and CD22	–	Combotox, Ricin-dgA
CD22	Epratuzumab	Moxetumomab pasudotox (PE38), Ricin-dgA, Inotuzumab ozogamicin (calicheamicin), ^131^I, ^186^Re, ^90^Y
CD25	Daclizumab	PE38, ricin-dgA, ^90^Y
CD25/CD122/CD132	–	Diptheria toxin A&B (Denileukin diftitox)
CD33	Lintuzumab	Gemtuzumab ozogamicin (calicheamicin), maytansinoid DM4, ^131^I, ^213^Bi, ^90^Y
CD45	Anti-CD45	^131^I
CD52	Alemtuzumab	–

*dgA, deglycosylated ricin A chain; PE, pseudomonas exotoxin A modified; TRAIL, tumor necrosis factor-related apoptosis-inducing ligand*.

### Unconjugated monoclonal antibodies

Unconjugated MoAbs have the potential to kill cells by direct and/or indirect mechanisms. Examples of direct effects include blockade of receptor/ligand interactions with interruption of growth signals, agonistic binding, and induction of apoptosis through initiation of a death-signal. Indirect killing is commonly immune-mediated, via complement-dependent cytotoxicity (CDC) and antibody-dependent cellular cytotoxicity (ADCC). In CDC, the Fc receptor of the MoAb fixes the complement protein C1q and triggers cell death by the activation of the complement cascade. ADCC is mediated by Fc interaction with and activation of effector cells such as NK cells and macrophages. Importantly, ADCC requires functional immune effector mechanisms, which may be deficient in the setting of childhood leukemia (Haining et al., 2005). It is likely that unconjugated MoAbs will have limited single agent efficacy in most cases of childhood leukemia, (Raetz et al., 2008a) although occasional CRs have been reported with MoAbs targeting CD52 (alemtuzumab) (Laporte et al., 2004), CD20 (rituximab) (Corbacioglu et al., 2003), and CD33 (lintuzumab) (Feldman et al., 2003). Additionally, activity was observed in some children with newly diagnosed mature B-cell ALL and NHL treated with single agent rituximab in a Phase II window study (Meinhardt et al., 2010).

Monoclonal antibodies have the potential to sensitize malignant cells to chemotherapy and/or radiotherapy (Wilson et al., 2009) or to induce the cell-surface expression of additional antigen targets (Rubinfeld et al., 2006), thus providing a rationale for combination therapy. Increased CD20 expression on ALL blasts has been observed during induction and corticosteroid therapy, which may make anti-CD20 directed therapy more effective following standard treatment (Dworzak et al., 2008). There is also the rationale for utilizing MoAbs in combination with adoptive cell therapies, for example to augment ADCC (Lang et al., 2004).

Based on the efficacy of unconjugated anti-CD20 MoAb (rituximab) in combination with chemotherapy for adults with NHL (Coiffier et al., 2002), studies have been designed to incorporate this agent into leukemia treatment. A trial conducted by investigators at the MD Anderson Cancer Center suggests that rituximab can be safely administered with the Hyper-CVAD regimen (cyclophosphamide, vincristine, adriamycin, and dexamethasone) and that adults with B-ALL might have improved leukemia-free survival in comparison to chemotherapy-only historical controls (Thomas et al., 2006, 2010). Results of a recent COG trial indicate that rituximab can be added to ifosfamide, carboplatin, and etoposide chemotherapy with an acceptable toxicity profile (Griffin et al., 2009). A pediatric trial of rituximab with chemotherapy for individuals with newly diagnosed B-ALL was recently completed through the COG (NCT00057811) with results pending.

Ofatumumab, is a fully humanized anti-CD20 MoAb that binds to a different epitope than rituximab and has stronger CDC activity in pre-clinical studies. A Phase II study of the Hyper-CVAD regimen in combination with ofatumumab as frontline therapy for patients with CD20 positive ALL is currently being conducted by investigators at MD Anderson Cancer Center (NCT01363128).

A study of the anti-CD22 MoAb epratuzumab in combination with standard chemotherapy was recently completed through the COG (NCT00098839). This trial included an initial cohort with an upfront epratuzumab monotherapy phase (Raetz et al., 2008a). The clinical activity of epratuzumab as a single agent was limited, with no complete or partial remissions observed in 15 patients, however it was well tolerated, both as a single agent and in combination with chemotherapy. In comparison to historical experience with the same chemotherapy backbone (Raetz et al., 2008b), the percentage of those individuals in CR after one block of induction who were MRD negative appeared favorable (Raetz et al., 2011).

### Conjugated monoclonal antibodies

The cytotoxicity of MoAbs can be increased by linkage to toxic moieties including chemotherapeutic agents, toxins, and radionuclides. Monoclonal antibody-based therapeutic agents armed with such potently cytotoxic compounds do not require active immune response mechanisms for activity and thus can be effective even in profoundly immunocompromised hosts.

#### Chemotherapeutic conjugates

Monoclonal antibodies have been conjugated to various active chemotherapy drugs. The first such agent to receive FDA approval was gemtuzumab ozogamicin (Mylotarg), which is an anti-CD33 MoAb linked to calicheamicin, a potent antitumor antibiotic. Clinical trials of gemtuzumab ozogamicin as a single agent and in combination with chemotherapy have been conducted in the setting of pediatric AML (Arceci et al., 2005; Aplenc et al., 2008). Approximately 30% of pediatric patients with relapsed and refractory AML respond to gemtuzumab ozogamicin as a single agent. Phase III trials designed to assess the efficacy of gemtuzumab ozogamicin in combination with chemotherapy in pediatric AML was initiated by the COG (AAML0531) with preliminary results suggestive of safety and efficacy in a pilot trial (Franklin et al., 2008). However, gemtuzumab ozogamicin was voluntarily withdrawn by its manufacturer in 2010 at the request of the FDA. This withdrawal was based on results of a randomized Phase III trial designed to assess the efficacy of gemtuzumab ozogamicin in combination with standard chemotherapy for adults with *de novo* AML conducted by the Southwest Oncology Group. There was no demonstrated benefit and increased rates of veno-occlusive disease and fatal toxicity were observed in the gemtuzumab ozogamicin arm (Petersdorf et al., 2013). Accrual to the COG AAML0531 trial was discontinued in 2010 when gemtuzumab ozogamicin was no longer available. Final analysis of results in those patients who had completed randomized therapy awaits longer follow-up. The Nordic Society of Paediatric Haematology and Oncology (NOPHO) conducted a randomized Phase III study of gemtuzumab ozogamicin as consolidation in children with AML (Hasle et al., 2012). There was no impact on the rate of relapse or survival in this study. A randomized, Phase III open-label study that employed fractionated low doses of gemtuzumab ozogamicin for adults with *de novo* AML (ALFA-0701) demonstrated improved event-free, relapse-free, and overall survival rates without an increase in fatal toxicity, suggesting that perhaps lower and fractionated dosing of this novel agent may be effective and more tolerable than previously tested regimens (Castaigne et al., 2012). Gemtuzumab ozogamicin has also been used to treat occasional cases of ALL with CD33 expression, with anecdotal reports of successful remission induction (Zwaan et al., 2003).

Inotuzumab ozogamicin is a CD22-targeted antibody conjugated to calicheamicin. A CR rate of 57% was observed in a Phase II trial in adults with relapsed and refractory ALL (Kantarjian et al., 2012). A randomized Phase III trial in adults (NCT01564784) is ongoing and a pediatric Phase I trial is planned.

#### Toxin conjugates

Bacterial and plant toxins such as *Diphtheria*, *Pseudomonas*, and ricin are highly potent cellular poisons. In addition, toxins are not susceptible to the multiple drug resistance gene-mediated mechanism of resistance to chemotherapy (Pastan et al., 2006). Immunotoxins are engineered proteins that consist of a MoAb-based targeting moiety linked to a toxin that upon internalization induces cell death by inhibiting protein synthesis. In general, clinical development of immunotoxins has been hindered by non-specific toxicities, immunogenicity, and production difficulties. At the NCI, a 38 kDa truncated derivative of *Pseudomonas* exotoxin A (PE38) has been used in the development of recombinant immunotoxins that target human differentiation antigens (FitzGerald et al., 2011). Serial modifications have reduced non-specific toxicities, increased stability, increased tissue penetration, and improved targeted cytotoxicity.

Recombinant anti-CD22 immunotoxins that contain the variable domains of the anti-CD22 MoAb RFB4 fused to a 38 kDa fragment of PE38 have been developed and tested at the NCI (Pastan et al., 2006). The first generation agent, BL22 (CAT-3888), was shown to be highly active in a Phase I study in adults with B-lymphoid malignancies (Kreitman et al., 2005, 2009, 2012). In a pediatric Phase I trial conducted at the NCI, BL22 had an acceptable toxicity profile and activity was seen at all dose levels (Wayne et al., 2010). A Phase I trial of a second-generation agent, moxetumomab pasudotox (HA22, CAT-8015), with higher CD22 binding affinity and increased pre-clinical activity (Mussai et al., 2010) is in progress (NCT00659425) with early results demonstrating CRs in children with chemotherapy refractory ALL (Wayne et al., 2011). Phase II trials of this agent are in development. Additionally, synergistic cytotoxicity was seen when moxetumomab pasudotox was combined with standard chemotherapy agents in pre-clinical studies (Ahuja et al., 2007) and a combination trial for pediatric ALL is planned.

Pediatric phase I trials of anti-CD19 immunotoxins have also been conducted (Uckun et al., 1999; Dinndorf et al., 2001), but these agents have not moved forward in clinical development. A Phase I trial of an immunotoxin mixture that targets CD19 and CD22 (Combotox) was performed in children with ALL with a reported CR rate of 21% (3 of 14 evaluable patients) (Herrera et al., 2009). Similar results were seen in a Phase I study in adults with ALL where a CR rate of 18% (3 of 17 subjects) and dose-limiting vascular-leak syndrome were observed (Schindler et al., 2011).

#### Radioimmunoconjugates

Monoclonal antibodies that target leukemia-associated or hematopoietic lineage antigens have been linked to radioactive isotopes, most commonly β-emitters (e.g., ^90^Yttrium, ^131^Iodine, ^186^Rhenium) and less frequently α-emitters (e.g., ^213^Bismuth) (Table 1). As these agents are concentrated in the marrow, they are associated with severe myelosuppression. Consequently, radioimmunotherapy has limited application in leukemia except as part of SCT regimens. ^131^Iodine conjugated-anti-CD45 MoAb in combination with non-myeloablative pre-transplant conditioning appeared to improve engraftment without a significant increase in toxicity in adults with advanced AML and high-risk myelodysplastic syndromes (Pagel et al., 2009). There have been very few pediatric trials of radioimmunoconjugates at least in part due to concerns for late effects of radiation.

#### Other conjugates

Other MoAb-based approaches have been employed to target leukemia-associated antigens. For example, anti-CD7 has been conjugated to tumor necrosis factor-related apoptosis-inducing ligand (TRAIL) fusion protein, which has potential utility in T-ALL (Bremer et al., 2005).

### Bi-specific monoclonal antibodies

Bi-specific antibodies have been designed to target both a leukemia-associated antigen and a surface receptor on immune effector cells such as T cells. The anti-CD19/anti-CD3ε bi-specific antibody (MT103, blinatumomab) is undergoing clinical development for the treatment of B-lineage hematologic malignancies in children and adults. In a Phase II trial for adults with ALL in MRD+ CR, this agent induced clearance of MRD and a high relapse-free survival rate in most patients (Topp et al., 2011, 2012a). In a subsequent exploratory phase II trial, blinatumomab was shown to induce CRs in adults with relapsed/refractory ALL (Topp et al., 2012b). A pediatric Phase I/II trial is currently in progress (NCT01471782). Importantly, bi-specific MoAbs require functional immune effector cells (e.g., T cells) for activity. Thus, it is possible that these agents may have increased activity following immune reconstitution after allogeneic SCT. Notably in that regard, three children with post-transplant relapse of ALL successfully achieved CRs with blinatumomab (Handgretinger et al., 2011).

## Cancer Vaccines

Initial studies of autologous cancer vaccines have been conducted in adult subjects. Subsequently, studies have been initiated in pediatric populations as well in the allogeneic setting.

### The immune response

T cells recognize antigens as small digested peptides displayed on the surface of antigen presenting cells (APCs) in the context of HLA. The generation of a successful T cell response requires the interaction of an appropriate T cell receptor with its cognate antigen/HLA complex as well as co-stimulation or a “second signal.” This second signal can be supplied in the form of an appropriate cytokine or by direct cell-to-cell interaction through accessory molecules.

### Leukemia-associated antigens

Numerous leukemia-associated antigens capable of inducing a T cell mediated immune response have been targeted with cancer vaccines. Examples include translocation fusion products, lineage-specific antigens, genes that are expressed aberrantly or in higher than normal levels, histocompatibility antigens and viral-associated antigens expressed by malignant cells. Various antigen sources have been used in vaccine trials. Peptides with defined sequences can be readily synthesized for clinical use and these have been employed most commonly.

### Peptide and full-length mRNA vaccines

Immunologic, clinical, and/or molecular responses have been observed in adults with leukemia treated with peptide vaccines targeting PR1 (Rezvani et al., 2008), WT1 (Mailander et al., 2004; Oka et al., 2004), and the *bcr/abl* breakpoint peptide (Jain et al., 2009). Importantly, the applicability of a peptide vaccine is restricted by both the specific antigen epitope and the HLA binding motif. Full-length mRNA vaccines represent a strategy to overcome such HLA and epitope restrictions. A clinical trial of a full-length WT1 mRNA dendritic cell vaccine in adults with AML demonstrated the potential utility of this approach in eliciting both clinical and immunological responses (Van Tendeloo et al., 2010).

### Dendritic cell-based vaccines and artificial antigen presenting cells

Dendritic cells are professional APCs that can be readily generated from peripheral blood monocytes and employed in cancer vaccines (Wong et al., 2001). Artificial APCs that express co-stimulatory molecules have also been engineered for use in cancer immunotherapy trials (Suhoski et al., 2007). Peptides, nucleic acids, proteins, and tumor lysates can be used to prime APCs to present leukemia-associated antigens. A clinical trial of a WT1 peptide allogeneic dendritic cell vaccine is being conducted at the NCI for children and adults with WT1-expressing hematologic malignancies and persistent or relapsed disease after SCT (NCT00608166). In this study, vaccines are administered along with DLI in attempt to augment the GVL effect, and immune responses have been observed in children with ALL treated on this trial (Shah et al., 2011).

### Whole tumor cell vaccines

Autologous and allogeneic tumor cell preparations can be employed as immunogenic sources in cancer vaccines in order to obviate the need to define the target antigen(s) and to avoid specific HLA allele restriction. The ready access to peripheral blood or bone marrow blasts makes this particularly applicable for leukemia vaccine trials. Blasts from children with B-precursor ALL can be rendered capable of presenting antigens by incubation with CD40 ligand and interleukin-4, thus, overcoming their ability to induce T cell anergy (Cardoso et al., 1996). However, a clinical trial using that approach was unsuccessful possibly due to both rapid disease progression and severe underlying immunosuppression (Haining et al., 2005).

### Adjuvants and combinations

The optimal immunotherapy reagents and regimens are expected to vary between diseases and individual cases. Critically important is the need to overcome the immune deficits associated with childhood leukemia and its treatment (Haining et al., 2005). Consequently, adjuvants are commonly employed to potentiate an immune response as part of immunotherapy trials (e.g., Montanide, GM-CSF, keyhole limpet hemocyanin). Toll-like receptors (TLRs) are a family of immune receptors that recognize structurally conserved molecules derived from microbes. TLR agonists can stimulate both innate and adaptive immune responses and have shown pre-clinical activity against ALL (Fujii et al., 2007). A pediatric Phase I trial of a TLR agonist for ALL and AML is in progress (NCT01743807).

## Conclusion

Although great strides have been made in the curative treatment of childhood leukemias, further progress will require novel therapeutic approaches (Wayne et al., 2008). Immunotherapy has the potential to mediate anti-leukemia activity with limited non-specific toxicities. As with chemotherapy, however, each immunotherapeutic approach has its own set of limitations, applicability, and risks and no single approach will be sufficient to treat all patients. Systematic study of multiple immune-based therapies will be necessary in order to define the optimal regimens and uses in the ongoing effort to develop new treatments for children, adolescents, and young adults with leukemia. Based on the potency of the GVL effect after SCT, it is predicted that methods that can induce and/or augment anti-leukemia immune responses hold great promise in the treatment of pediatric leukemia.

## Conflict of Interest Statement

The authors declare that the research was conducted in the absence of any commercial or financial relationships that could be construed as a potential conflict of interest.
